# Negative Predictive Value of the Rapid Test Ag 2019-nCoV During the Predominance of Omicron over the Delta Variant and Implications in the Emergency Department

**DOI:** 10.1007/s42399-022-01294-y

**Published:** 2022-09-27

**Authors:** Barbara Fyntanidou, Georgios Meletis, Sofia Gkarmiri, Ioanna Gkeka, Areti Tychala, Lemonia Skoura

**Affiliations:** 1 Department of Emergency Medicine, AHEPA University Hospital, S. Kiriakidi str. 1, 54636 Thessaloniki, Greece; 2 Department of Microbiology, AHEPA University Hospital, Thessaloniki, Greece

**Keywords:** COVID-19, SARS-CoV-2, Rapid test, Omicron, Delta

## Abstract

The high prevalence of asymptomatic patients infected with SARS-CoV-2 during the pandemic peaks and the common occurrence of in-hospital transmission urges the need for SARS-CoV-2 testing before admission of all patients with non-COVID-related symptoms. RT-PCR testing however is costly, time-consuming, and increases the length of stay in the emergency department. For the aforementioned reasons, we propose that the admission of non-suspected COVID-19 patients to the appropriate department should be based on the sole use of the rapid test result. In order to assess the safety of this suggestion, we assessed the negative predictive value of our rapid antigen tests that was calculated at 96.38%. This value was considered acceptable and the proposed strategy was applied in our hospital improving the overall turnaround times. However, since various rapid tests may perform differently, we propose that hospitals assess their own methodologies before implementing our proposal.

During the first months of the COVID-19 pandemic, there was a considerable worldwide decline in emergency department (ED) visits for non-COVID-related symptoms, resulting in a decrease in the usual overcrowding of the ED [1]. Lockdowns, government restrictions, social distancing, telephone or online medical consultations, reduction of non-emergency hospital admissions and procedures, the fear of the unknown, and the untreatable prevented the ED overcrowding [1, 2].

As the new normal settles and the world resumes “business as usual,” we observe an expected rise in ED visits for non-COVID-related symptoms. However, the bifurcation of the pathway to emergency health services and resources for suspected and non-suspected COVID-19 patients is still applied for the safety of all involved, resulting in further overcrowding and increased length of stay in the ED.

Due to the common occurrence of in-hospital transmission of severe acute respiratory coronavirus 2 (SARS-CoV-2), especially during the pandemic peaks and the high prevalence of asymptomatic patients infected with SARS-CoV-2, our hospital initially implemented an obligatory testing for SARS-CoV-2 with real-time polymerase chain reaction (RT-PCR) before admission of all patients with non-COVID-related symptoms. The aim was to ensure the safety of the hospital environment for both the patients and the staff, to reduce the hospital-acquired COVID-19 as well as the need for in-hospital transfer of patients and quarantining of hospital departments.

RT-PCR testing however is costly and time-consuming. Its use in the emergency setting for the admission of non-suspected for COVID-19 patients increases the risk and duration of potential exposure in the finite and overcrowded emergency department and the length of stay in the ED (which is linked to increased morbidity, mortality, and longer duration of hospital stays [3–5]) and influences the quality and timeliness of the emergency health services provided.

Rapid antigen lateral flow assays on the other hand are less expensive, easy to perform, and provide results in a few minutes. It is well known however that they are less reliable than RT-PCR. Their sensitivity and specificity have been extensively studied and are commonly within the range of 85–92% and 95–99%, respectively [6]. Their limit of detection is lower than that of molecular methods [7] and their results depend also on the timing of testing regarding the onset of symptoms, with positive results being more probable to occur in already symptomatic individuals [8].

In an effort to improve the quality and timelines of our ED services in the context of an ongoing pandemic, we suggest that the admission of non-suspected COVID-19 patients to the appropriate department should be based on the sole use of the rapid test result. In order to assess the safety of this suggestion, we calculated the negative predictive value of our rapid antigen tests.

We retrospectively reviewed 636 negative rapid test results (Rapid Test Ag 2019-nCoV, ProGnosis Biotech S.A., Larissa, Greece, manufactured to detect the SARS-CoV-2 nucleocapsid protein) of nasopharyngeal specimens collected in our emergency department between November 1, 2021, and January 21, 2022, that were tested also by RT-PCR on the same day using the NeuMoDx SARS-CoV-2 or the Abbott RealTime SARS-CoV-2 assay. The Rapid Test Ag 2019-nCoV has a sensitivity of 85.5% and a specificity of 99.8% [9]. From November 1 to mid-December 2021, Delta was the only variant present in our hospital. On December 20, 2021, the Omicron variant emerged and prevailed within the range of 2 weeks. Delta however continued to be detected in lower rates. The national prevalence of COVID-19 was 0.67% on November 1, 2021, and 3.15% on January 21, 2022. During this period, the maximum prevalence was 4.43% on January 9, 2022 (https://www.worldometers.info/coronavirus/country/greece/). Among the tested specimens, 23 had a positive PCR result and were considered false negatives (Table 1). The negative predictive value of the rapid antigen test was calculated at 96.38%. Even though the cycle threshold values (Cts) of the false negative specimens varied and were not all higher than the positivity limits applied in our laboratory (≤ 30 for NeuMoDx SARS-CoV-2 and ≤ 27 for Abbott RealTime SARS-CoV-2), the negative predictive value of our rapid antigen test was considered acceptable and the proposed strategy was applied in our hospital with satisfactory results. More precisely, we propose rapid testing for all patients at a pre-triage office. Patients with negative test results proceed to a “clean” non-COVID ED. This way, the COVID ED is not burdened with additional patients that would delay the already complicated procedures that have to be followed for COVID patients. This decision improved the overall turnaround times because rapid testing is performed on site and lasts a few minutes whereas RT-PCR results may need from 2 up to 8 h depending on the workload and the laboratory workflow.

**Table 1:** Ct values of 23 false negative Rapid Test Ag 2019-nCoV results on NeuMoDx SARS-CoV-2 or Abbott RealTime SARS-CoV-2 Assay. *Ct*, cycle threshold; *N*, nucleocapside gene; *Nsp2*, non-structural protein 2 gene; *RdRp*, RNA-dependent RNA polymerase gene

Test ID	NeuMoDx N Ct	NeuMoDx Nsp2 Ct	Abbott RealTime RdRp and N Ct
287.937	12.85	14.24	
228.075	17.08	17.7	
1428	17.57	18.77	
287.927	15.01	16.8	
4152	32.84	31.14	
9029	32.92	32.24	
12.124	15.5	16.61	
9401	20.84	21.5	
228.020	12.64	13.71	
254.234	13.5	15.2	
2992	14.67	15.07	
1536	26.72	27.74	
4406	25.45	25.88	
261.655	31.35	31.2	
9176	31.38	30.76	
262.074	25.83	26.41	
11.997	32.21	31.75	
268.751	26.42	28	
242.395	16.44	17.66	
6709			3.28
266.424	29.27	29.68	
262.218	20.34	20.78	
16.947	26.61	27.95	

These results however cannot be generalized, since various lateral flow assays may perform differently. We therefore propose that hospitals assess their own methodologies before implementing our strategy.

## Data Availability

Not applicable.
